# Absence of the Proper Hepatic Artery Detected on Cadaveric Dissection

**DOI:** 10.7759/cureus.79957

**Published:** 2025-03-03

**Authors:** George Tsakotos, George Triantafyllou, Orestis Lyros, Ioannis Paschopoulos, Maria Piagkou

**Affiliations:** 1 Department of Anatomy, School of Medicine, Faculty of Health Sciences, National and Kapodistrian University of Athens, Athens, GRC; 2 Fourth Department of Surgery, Attikon University Hospital, School of Medicine, National and Kapodistrian University of Athens, Athens, GRC

**Keywords:** cadaveric dissection, coeliac trunk, hepatic artery system, proper hepatic artery, variation

## Abstract

The coeliac trunk (CeT) and hepatic arterial system exhibit significant anatomical variability, which holds substantial clinical and surgical relevance. This cadaveric case describes an aberrant branching pattern of the CeT discovered during the routine anatomical dissection of a 78-year-old female cadaver. Notably, the proper hepatic artery (PHA) was absent, with the left hepatic artery (LHA) arising from the common hepatic artery (CHA) before its bifurcation into the right hepatic artery (RHA) and gastroduodenal artery (GDA). In addition, the inferior phrenic arteries (IPAs) originated from a common trunk arising from the CeT. This vascular configuration deviates from the CHA trifurcation pattern, which has been previously documented in the literature but remains infrequently reported. Given the critical role of the hepatic arterial system in surgical procedures, including hepatectomy, liver transplantation, and interventional radiology, recognizing such anatomical variations is paramount to prevent intraoperative complications. This report underscores the importance of detailed preoperative imaging and thorough anatomical knowledge in optimizing surgical outcomes and patient safety.

## Introduction

The major branches of the abdominal aorta, which supply the abdominal organs, are the coeliac trunk (CeT), superior mesenteric artery (SMA), and inferior mesenteric artery. The typical anatomy of the CeT is described as a trifurcation into the left gastric artery (LGA), splenic artery (SA), and common hepatic artery (CHA). The CHA bifurcates into the gastroduodenal artery (GDA) and proper hepatic artery (PHA). The PHA gives off the left hepatic (LHA) and right hepatic arteries (RHA) that provide the arterial supply to the liver [1], while the RHA also provides the cystic artery (CA) for the supply of the gallbladder [2].

Frequently, these vessels can present anatomic variants that are recorded during cadaveric dissection, imaging techniques, or intraoperatively. CeT variants were calculated with an overall pooled prevalence of 10.53% [3]. One of the most common variants is the presence of the inferior phrenic arteries originating from the CeT [4]. Moreover, extensive research has been performed on the hepatic arterial system, including the variant origin or the accessory forms of the LHA and RHA [5]. The morphological variability of these arteries has substantial surgical significance because alterations of the typical anatomy can change the surgeon’s approach or lead to iatrogenic injuries.

Herein, we describe an aberrant anatomy of the CeT branches identified incidentally during educational dissection. The morphological variability and the clinical significance will be further discussed.

## Case presentation

During routine anatomical dissection for educational and research purposes of a 78-year-old female cadaver, the abdominal arteries presented with interesting variants. The cadaver was donated to the Department of Anatomy (School of Medicine, National and Kapodistrian University of Athens) through the “Body Donation Program” [6]. Dissection of the abdominal cavity was performed with an incision along the linea alba. The upper abdominal organs were dissected and mobilized to expose the abdominal vessels. An electronic caliper (Mitutoyo Corporation, Kawasaki-shi, Kanagawa, Japan) was used for the measurements. Each measurement was repeated twice with an accuracy of up to 0.1 mm.

**Figure 1:**
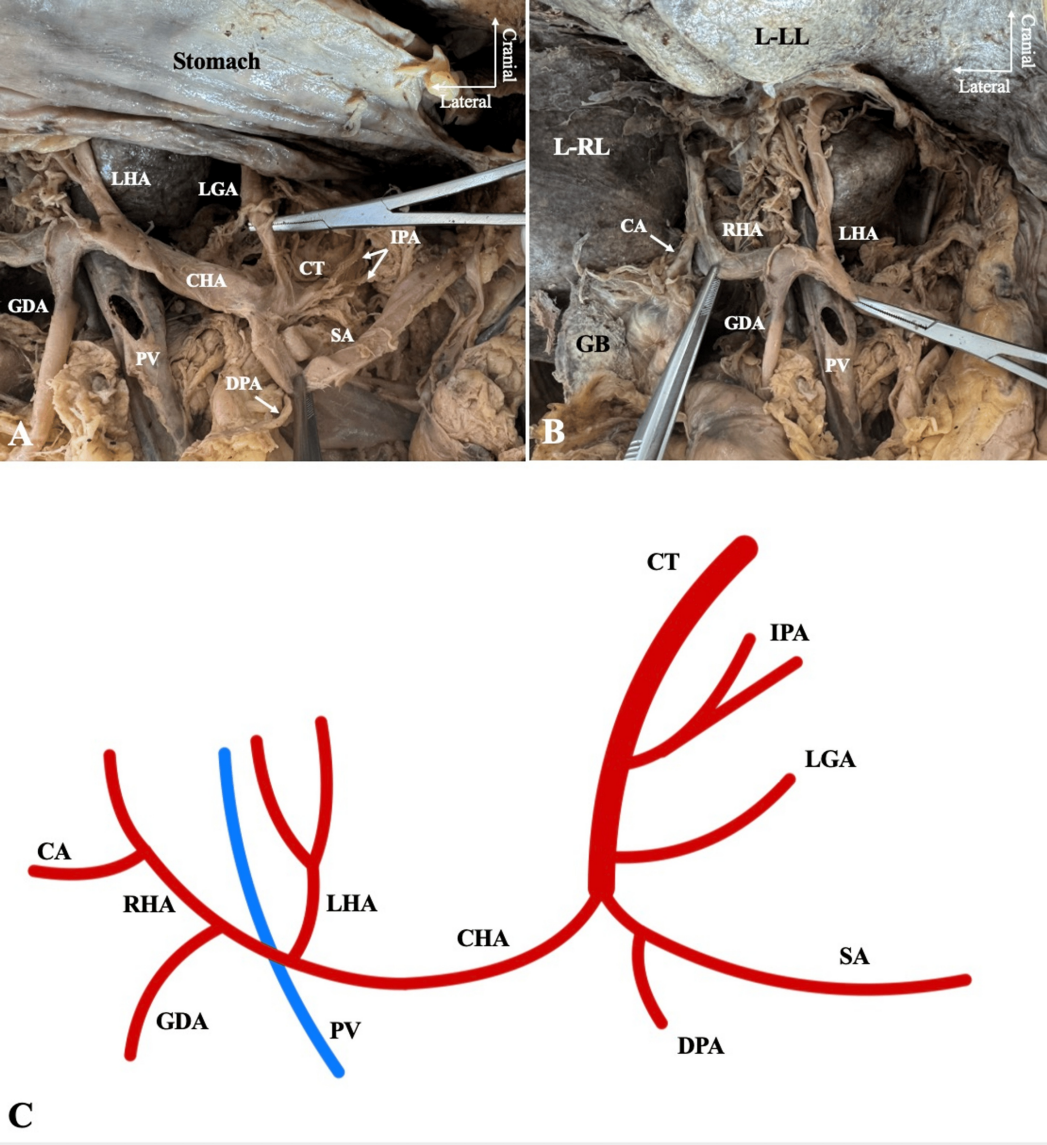
The abdominal arteries of the 78-year-old female cadaver. (A) The celiac trunk (CT) that gave off a common trunk for the inferior phrenic arteries (IPA), the left gastric artery (LGA), the splenic artery (SA) and the common hepatic artery (CHA). The dorsal pancreatic artery (DPA) emanated from the SA. (B) The common hepatic artery gave off the left hepatic artery (LHA), and then, it bifurcated into the gastroduodenal artery (GDA) and the right hepatic artery (RHA) that gave off the cystic artery (CA). (C) A schematic representation of the identified anatomy. L-LL: left lobe of the liver, L-RL: right lobe of the liver, GB: gallbladder, PV: portal vein

The CeT was identified as the first branch of the abdominal aorta. First, it gave off a common trunk for the IPAs. Second, the LGA was originated, and then, it bifurcated into the SA and CHA. After 22.8 mm, the SA gave off the dorsal pancreatic artery (DPA). The LHA was originated from the CHA (57.6 mm after its origin), and it had an early bifurcation into two branches. The CHA, 12.6 mm after the LHA origin, bifurcated into the GDA and the RHA. Therefore, the PHA was absent (Figure 1). Lastly, the GDA gave off the right gastric and right gastroepiploic arteries.

## Discussion

The most important finding of the present case was the absence of the PHA. In the current literature, this absence is an overlooked variant that is not frequently described when the hepatic arterial system is investigated. Song et al. [7] evaluated the hepatic arterial system in 5002 angiograms, and they did not report the PHA's absence. Ekingen et al. [8] described in detail the PHA variants, and they observed the absence of the PHA with CHA trifurcation into the LHA, RHA, and GDA (8.94% of cases). Recently, Manta et al. [7] also reported the CHA trifurcation (PHA absence) coexisting with hexafurcated CeT in two patients. However, in the current case, the LHA was originated, and after 12.6 mm, the CHA was bifurcated into the RHA and GDA. Therefore, this pattern is different from the CHA trifurcation (PHA absent) described in the current literature. To the authors’ knowledge, one similar case was recorded by McDaniel and Frank [9] during a routine anatomical dissection.

The CeT and hepatic arterial system (CHA, LHA, and RHA) have an exceptionally high morphological variability, with multiple studies investigating their variations. The most common variant of the CeT is the presence of the IPA or DPA originating from the trunk. The IPAs originate from a common trunk in 24.2% of the population, while the most common variant origin is the CeT in 41.3% [4]. Other origins are described rarely, with an overall prevalence of 1% [4]. Moreover, the DPA origin from the CeT was estimated with a pooled prevalence of 11.9% [10]. The most common origin was the SA (similar to the current case) in 37.6%, while the SMA origin was calculated in 23.9% [10]. Scarcely in the literature, CeT variations formatting a hexafurcated or heptafurcated trunk were described [7,11]. Furthermore, Cirocchi et al. [5] observed the pooled prevalence of an aberrant LHA (replaced or accessory) in 13.52%. The replaced LHA was estimated at 8.26%, while the accessory LHA was at 5.55% [5]. The most common origin of a replaced or accessory LHA is described as the LGA [5]. Rare cases have also been described. Natsis et al. [12] observed the CHA absence and the coexistence of replaced PHA and GDA during cadaveric dissection.

Nowadays, the major abdominal vessels can be easily visualized through computed tomography or magnetic resonance angiograms and provide anatomical details preoperatively. During these techniques, a vast majority of variants are described. An absence of the PHA and the aberrant LHA arising from the CHA before its bifurcation, observed in the current case, significantly changes the hepatic vasculature. Knowledge of the hepatic arterial system and the associated lymphatics are of paramount importance during hepatectomy and the formation of proper anastomosis with the recipient’s vasculature during transplant. Aberrant and/or accessory branches, as well as the absence of the typical vessels (similar to the current case), significantly alter the anatomy of the area and can precipitate surgical difficulties [9]. Nevertheless, the arterial anatomy is also essential for interventional radiologists who guide catheters through the vessels to target different tissues for embolization or drainage [9]. Thus, preoperative and intraoperative imaging and proper identification of the vessels are adequate. In addition, the anatomy of the CeT should be investigated for surgery of the stomach, pancreas, spleen, liver, and biliary tree because all these structures receive their arterial supply from the CeT branches [3]. A computed tomography angiography (CTA) gives important vascular information for the CeT and hepatic arterial system to surgeons prior to operations in the region. Aberrant or accessory vessels are easily identified, as well as the absence of major vessels such as the PHA (described in the current case) or the CeT. In our previous meta-analysis, we calculated the pooled prevalence of the CeT absence in 0.43%, demonstrating a rare variation [3].

## Conclusions

In the current cadaveric report, we described the PHA absence with the LHA arising from the CHA before its bifurcation. Although the PHA absence with CHA trifurcation into the LHA, RHA, and GDA has been described in the available literature infrequently, the present arterial pattern was reported only once. Nevertheless, the IPAs originated via a common trunk from the CeT. Precise knowledge of the CeT and hepatic arterial system is of paramount importance for surgeons and interventional radiologists.
